# β-Cell Mitochondrial Dysfunction: Underlying Mechanisms and Potential Therapeutic Strategies

**DOI:** 10.3390/cells14231861

**Published:** 2025-11-26

**Authors:** Radwan Darwish, Yasmine Alcibahy, Ghena Abu-Sharia, Alexandra E. Butler

**Affiliations:** 1 School of Medicine, Royal College of Surgeons in Ireland-Medical University of Bahrain, Busaiteen P.O. Box 15503, Bahrain; 22200493@rcsi-mub.com (R.D.); 21201313@rcsi-mub.com (Y.A.); 2 College of Medicine, Alfaisal University, Riyadh 11533, Saudi Arabia; gabusharia@alfaisal.edu; 3 Research Department, Royal College of Surgeons in Ireland-Medical University of Bahrain, Busaiteen P.O. Box 15503, Bahrain

**Keywords:** β-cell, mitochondria, mitochondrial dysfunction, mitophagy, diabetes

## Abstract

Mitochondria are essential for β-cell function, coupling glucose metabolism to ATP production and insulin secretion. In diabetes, β-cell mitochondrial dysfunction arises from oxidative stress, impaired quality control and disrupted dynamics, leading to reduced oxidative phosphorylation, defective insulin release and progressive cell loss. Key transcriptional regulators link genetic susceptibility to mitochondrial dysfunction in both type 1 diabetes mellitus (T1DM) and type 2 diabetes mellitus (T2DM). These disruptions impair mitophagy, mitochondrial translation and redox homeostasis. Therapeutic strategies that restore mitochondrial function, including mitophagy enhancers, mitochondrial antioxidants, and transcriptional regulators, have shown potential in preserving β-cell integrity. As mitochondrial failure precedes β-cell loss, targeting mitochondrial pathways may represent a critical approach to modifying diabetes progression.

## 1. Introduction

Pancreatic β-cells are highly specialized for glucose-stimulated insulin secretion (GSIS) and rely critically on mitochondrial metabolism to translate glucose sensing into exocytosis. Glucose enters β-cells and is metabolized via glycolysis to pyruvate, which fuels the tricarboxylic acid (TCA) cycle and oxidative phosphorylation. In the mitochondria, oxidation of pyruvate generates NADH and FADH_2_, driving electron transport, ATP synthesis and the generation of metabolic coupling factors (such as ATP, GTP, NADPH and intermediate metabolites) that trigger insulin granule exocytosis [1,2,3,4]. Calcium uptake into mitochondria further stimulates TCA cycle dehydrogenases, amplifying ATP production and insulin release [5]. β-cell mitochondria also supply reducing equivalents (NADPH) and ATP to the endoplasmic reticulum (ER) to support proinsulin folding and maturation [6,7]. Indeed, the ER and mitochondria operate in redox balance: proinsulin oxidative folding in the ER requires NADPH from mitochondria, and mitochondrial function depends on ER redox homeostasis [8]. In short, β-cells funnel glucose-derived carbons and energy through mitochondria for insulin secretion, and any disruption of mitochondrial ATP or coupling-factor production impairs GSIS [9]. The interplay between β-cell mitochondrial bioenergetics and glucose metabolism is therefore not only important, but necessary, for normal insulin secretion and glucose homeostasis [10].

Although oxidative phosphorylation (OXPHOS)-derived ATP has long been viewed as the principal metabolic signal driving GSIS, emerging data challenge this traditional model. Recent studies suggest that glycolytic ATP production and cytosolic ATP gradients may serve as key coupling factors for insulin exocytosis, particularly during the initial glucose response. At the plasma membrane, local pyruvate Kinase (PK), converts phosphoenolpyruvate (PEP) to ATP, thereby closing K_ATP_ channels and causing Ca^2+^ influx independently of mitochondrial oxidative phosphorylation. Genetic and pharmacologic activation of PK increases cytosolic ATP/ADP and insulin secretion [11,12]. Furthermore, direct electrophysiological recordings have demonstrated the presence of a plasma membrane associated glycolytic metabolon that locally channels substrates through phosphofructokinase and pyruvate kinase to generate ATP and modulate K_ATP_ ATP channel activity, reinforcing the concept of compartmentalized glycolytic ATP signaling in β-cell excitability [13]. This has led to a growing debate over whether OXPHOS-derived ATP or glycolytically generated ATP plays the dominant role in β-cell stimulus–secretion coupling.

However, this compartmentalized glycolytic ATP model does not fully negate the established role of mitochondrial metabolism in sustaining insulin secretion. While glycolytic ATP may cause K_ATP_ closure and membrane depolarization, mitochondrial oxidative phosphorylation remains essential for sustaining the secretory response through the continuous provision of ATP, NADPH, and anaplerotic intermediates that fuel the TCA cycle [14,15]. In fact, studies using mitochondrial inhibitors demonstrate that β-cell depolarization may occur in the absence of OXPHOS, but the second phase of insulin release collapses when mitochondrial metabolism is impaired, emphasizing a sequential interplay between glycolytic initiation and mitochondrial amplification [16].

These findings suggest that β-cell bioenergetics are spatially and temporally compartmentalized rather than mutually exclusive, glycolytic ATP may act locally at the plasma membrane, while mitochondrial ATP and coupling factors regulate global energy homeostasis and redox signaling [17]. Thus, the current debate reflects not a replacement of the mitochondrial model, but a refinement toward a dual-pathway framework in which both glycolytic and oxidative ATP production cooperate to support GSIS. Understanding this integrated metabolic crosstalk is crucial, as mitochondrial dysfunction in diabetes likely disrupts not only oxidative ATP supply but also the downstream coordination of glycolytic flux and cellular excitability.

Further, despite progress toward a dual-pathway model, several key mechanistic questions remain unresolved, and merit targeted experimental investigation. First, what is the relative spatiotemporal contribution of glycolytic versus mitochondrial ATP to K_ATP_ channel closure in intact islets under physiological glucose oscillations? This question could be addressed using genetically encoded ATP sensors with subcellular targeting (e.g., plasma membrane-targeted versus mitochondria-targeted PercevalHR or iATPSnFR2) combined with simultaneous electrophysiology and live-cell imaging in human islets [18,19]. Such experiments would reveal whether local ATP gradients at the plasma membrane are sufficient to fully explain first-phase insulin secretion or whether mitochondrial ATP contributes even at this early stage. Second, how do different glucose concentrations (5 mM vs. 10 mM vs. 20 mM) shift the relative dependence on glycolytic versus mitochondrial pathways? Dose–response studies using selective inhibitors (e.g., UK5099 to block mitochondrial pyruvate transport, or oligomycin to inhibit ATP synthase) alongside glycolytic enzyme modulators (e.g., TEPP-46 to activate PKM2) would delineate the glucose concentration thresholds at which each pathway becomes rate-limiting [11,20]. Third, does the glycolytic metabolon at the plasma membrane physically interact with mitochondria via mitochondria-associated ER membranes (MAMs) or other contact sites? High-resolution proximity labeling (e.g., TurboID or APEX2 targeted to plasma membrane K_ATP_ channels or glycolytic enzymes) followed by mass spectrometry could identify whether mitochondrial proteins participate in plasma membrane metabolic signaling complexes [21]. Fourth, can mitochondrial dysfunction be rescued by enhancing glycolytic ATP production, or does loss of mitochondrial coupling factors (NADPH, GTP, anaplerotic substrates) represent an insurmountable barrier? β-cell-specific overexpression of plasma membrane-targeted pyruvate kinase isoforms in mitochondrial transcription factor A (*TFAM*) knockout or mtDNA-depleted models would test whether amplifying local glycolytic ATP can compensate for mitochondrial failure [12,22]. Finally, how does chronic hyperglycemia reprogram the balance between glycolytic and mitochondrial ATP signaling in human T2DM islets? Single-cell metabolic flux analysis using ^13^C-glucose tracing combined with single-cell RNA-seq and ATP imaging in islets from diabetic donors could reveal whether the shift toward glycolytic dependence represents a compensatory adaptation or a maladaptive metabolic rewiring [23]. Resolving these questions will require integrating cutting-edge imaging, metabolomics, and genetic tools in physiologically relevant β-cell models and will ultimately determine whether therapeutic strategies should aim to restore mitochondrial function, enhance glycolytic capacity, or rebalance both pathways.

That said, under physiologic conditions, β-cell mitochondria maintain an optimal balance between energy production and reactive oxygen species (ROS) generation. Low physiological levels of ROS can act as signalling molecules, but excessive ROS from mitochondrial respiration or nutrient excess induces oxidative damage to mitochondrial DNA, proteins and lipids. β-cells have relatively low antioxidant defences, making them especially vulnerable to oxidative stress. Chronic nutrient overload (glucotoxicity and lipotoxicity) leads to mitochondrial overwork and ROS accumulation. In vitro studies show that under conditions of high glucose and/or fatty acids, β-cell apoptosis is driven by mitochondrial ROS: antioxidants can prevent β-cell apoptosis in this context, implying a causal role of mitochondria in the damage [1]. Disruption of the mitochondrial membrane potential under stress further impairs ATP synthesis, blunting GSIS. Moreover, ER stress and chronic misfolding of proinsulin also perturb mitochondrial redox balance. ER stress releases Ca^2+^ into the cytosol and generates additional ROS, overloading mitochondria and creating a vicious cycle of dysfunction [24]. Thus, mitochondrial dysfunction in β-cells, whether due to intrinsic defects or extrinsic stress, leads to defective insulin secretion and β-cell failure.

## 2. Mitochondrial Dynamics, Quality Control, and Mitophagy

Mitochondrial morphology and turnover are tightly regulated to match metabolic needs. Mitochondria constantly undergo fission and fusion: fusion (mediated by Mitofusin 1 and 2 (MFN1/2) and Optic atrophy 1 (OPA1)) allows mixing of mitochondrial contents, whereas fission (mediated by Dynamin-related protein 1 (Drp1)) helps segregate damaged segments [25]. Under stress, β-cells tend to show fragmented mitochondria, reflecting enhanced fission or impaired fusion. This fragmentation affects mitochondrial function and can also act as a signal for initial general autophagy, allowing the cell to degrade damaged organelles and maintain homeostasis [26,27,28]. For example, exposure to high glucose or cytokines downregulates Mfn2 [29] and upregulates Drp1 in β-cells, promoting fragmentation and isolating damaged portions for autophagic removal. Fragmented mitochondria are often less efficient in ATP production [30,31,32].

While excessive fragmentation is indeed a hallmark of stressed β-cells, it is important to note that mitochondrial fission also serves essential physiological roles [33]. Recent evidence indicates that β-cell mitochondrial fission dynamically responds to metabolic changes such as hyperglycemia, starvation, or ATP synthase inhibition, highlighting that fragmentation can occur as a physiological adaptation rather than solely under stress [27]. Similarly, a study using β-cell-specific Drp1 knockout mice exhibited highly fused mitochondria, abnormal Ca^2+^ handling, and blunted GSIS despite normal oxygen consumption, indicating that fission is required for physiological responsiveness to glucose as well [34].

Mitophagy, the selective autophagy of mitochondria, is a critical quality-control mechanism in β-cells. Under normal conditions, basal mitophagy helps eliminate old or mildly dysfunctional mitochondria, maintaining a healthy pool [35,36,37]. Under stress, enhanced mitophagy clears severely damaged organelles. The canonical PINK1-Parkin pathway initiates mitophagy: PINK1 accumulates on depolarized mitochondria and recruits Parkin, which ubiquitinates outer membrane proteins to tag the organelle for autophagosome engulfment [38,39]. Further, β-cells exposed to diabetogenic cytokines (IL-1β, IFN-γ) upregulate mitophagy as a compensatory response to nitrosative/oxidative damage [33]. Mitophagy-deficient β-cells in the islets of Mt Keima mice accumulated fragmented, dysfunctional mitochondria and exhibited worsened hyperglycemia under stress. Further, overexpression of *CLEC16A* [40], a T1DM susceptibility gene and known mitophagy regulator, reduced β-cell apoptosis [33]. Thus, mitophagy is cytoprotective, removing injured mitochondria, preventing cytosolic release of proapoptotic factors, and sustaining ATP supply. Other studies have also previously implicated macroautophagy in the cytoprotection of β-cells [41,42].

A β-cell-specific ubiquitin-dependent complex plays a pivotal role in regulating mitophagy. The *CLEC16A* gene encodes an E3 ubiquitin ligase that partners with *NRDP1* and the deubiquitinase *USP8* to form a tripartite complex essential for mitochondrial quality control [40]. CLEC16A facilitates non-degradative ubiquitin tagging of itself and NRDP1, stabilizing the complex and creating a scaffold for the recruitment of mitophagy effectors such as Parkin [40]. Disruption of this complex, either pharmacologically, as with lenalidomide [43], or metabolically, as seen under glucolipotoxic stress [44,45], impairs mitophagy, diminishes mitochondrial respiration, and reduces insulin secretion. These findings link dysfunction of the CLEC16A-NRDP1-USP8 axis to β-cell failure and highlight the importance of ubiquitin signalling in maintaining mitochondrial integrity in diabetes.

In 2025, Walker et al. [46] uncovered a conserved mitochondrial retrograde signaling pathway that links mitochondrial quality control failure to loss of cellular maturity across metabolic tissues, including pancreatic β-cells. Using complementary genetic models targeting mitophagy (*Clec16a* knockout), mitochondrial genome integrity (*Tfam* knockout), and mitochondrial fusion (*Mfn1/2* double knockout), they demonstrated that defects in these pathways converge on electron transport chain (ETC) and oxidative phosphorylation (OXPHOS) impairment, triggering activation of the integrated stress response (ISR). This was characterized by eIF2α phosphorylation, ATF4 stabilization, and induction of canonical ISR effectors such as *Ddit3*/CHOP, *Atf3*, *Cebpβ*, and *Gdf15*, independent of ER stress. In β-cells, this retrograde signaling led to downregulation of key identity genes (*Ins2*, *MafA*, *Ucn3*, *Slc2a2*/GLUT2) and induction of dedifferentiation markers (*Aldh1a3*, *Cd81*), without evidence of apoptosis or progenitor reversion. Mechanistically, early ETC/OXPHOS defects, not oxidative stress, were shown to initiate ISR activation, as restoration of NADH oxidation or treatment with the OXPHOS enhancer (–)-epicatechin suppressed ISR signaling. Single-nucleus ATAC-seq further revealed loss of chromatin accessibility at β-cell identity loci and enrichment of ATF4 and BACH2 motifs, suggesting chromatin remodeling as a downstream consequence of mitochondrial distress. Crucially, pharmacologic inhibition of the ISR with ISRIB restored β-cell mass, *Ucn3* expression, and glucose tolerance, confirming that mitochondrial stress–induced dedifferentiation is reversible. Collectively, these findings position mitochondrial integrity as a primary determinant of β-cell identity and reveal the ISR as a key therapeutic target for preserving β-cell maturity under metabolic stress.

While these findings establish a retrograde signaling route by which mitochondrial dysfunction can reprogram β-cell fate, anterograde regulators (e.g., FOXO1, PGC1α, PRDM16, ERRγ, and PDX1) have been shown to influence mitochondrial metabolism and maturity. However, their effects are tissue-specific and do not uniformly regulate both identity and mitochondrial function [47,48,49,50,51]. For example, FOXO1 promotes β-cell identity but enhances brown adipose tissue (BAT) differentiation [52,53], while PGC1α is critical for BAT but dispensable in β-cells [49,54]. These limitations in tissue specificity and pathway overlap have made it difficult to establish whether mitochondrial regulation alone is sufficient to govern cellular maturity.

**Figure 1:**
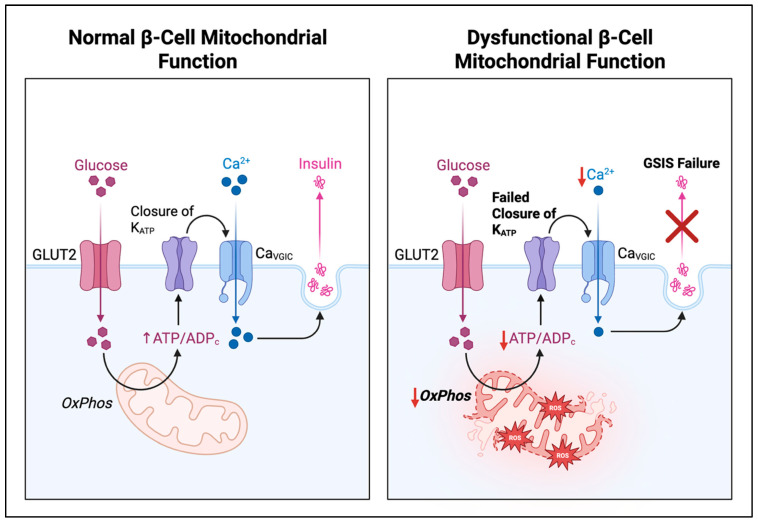
Contrasting Normal and Dysfunctional Mitochondrial Regulation of Glucose-Stimulated Insulin Secretion in Pancreatic β-Cells with Efficient Oxidative Phosphorylation and ATP Production Supporting Insulin Release in Health and Mitochondrial Fragmentation Impaired Mitophagy Oxidative Stress and Secretory Failure in Diabetes. Main Differences: (1) In normal β-cells, mitochondria carry out efficient oxidative phosphorylation, producing a high ATP/ADP ratio (black arrows indicate the sequence of metabolic and signaling events) whereas, in dysfunctional β-cells, oxidative phosphorylation is reduced, ATP generation is impaired and reactive oxygen species accumulate (red arrows indicate impaired or diminished processes). (2) In normal β-cells, glucose uptake (pink arrows) leads to a rise in ATP levels leads to the closure of K_ATP_ channels and subsequent membrane depolarization while, in dysfunctional β-cells, insufficient ATP prevents K_ATP_ channel closure and depolarization fails to occur. (3) In normal β-cells, depolarization activates voltage-gated calcium channels, allowing calcium influx (blue arrows), whereas, in dysfunctional β-cells, calcium entry is reduced due to the lack of membrane depolarization, leading to insulin release failure (red cross ‘X’ indicates process inhibition or failure). GLUT2: glucose transporter 2; K_ATP_: ATP-sensitive potassium channel; ATP: adenosine triphosphate; ADP: adenosine diphosphate; Ca_VGIC_: voltage-gated calcium channel; OxPhos: oxidative phosphorylation; GSIS: glucose-stimulated insulin secretion.

Overall, diabetic β-cells exhibit a downward spiral: nutrient and inflammatory stresses increase mitochondrial ROS and Ca^2+^ load, triggering fragmentation and mitophagy. If clearance is insufficient or signalling is defective, dysfunctional mitochondria accumulate, bioenergetics collapse, and apoptotic pathways are activated (e.g., cytochrome-c release, caspase-3). The net result is impaired GSIS and loss of β-cell mass, hallmarks of both T1DM and T2DM pathogenesis [55,56]. This is captured in Figure 1 below.

## 3. Genetic and Transcriptional Regulators of β-Cell Mitochondria

Nuclear transcription factors and genetic variants influence mitochondrial biogenesis and maintenance. First, transcription factor PDX1, a key β-cell differentiation gene, directly controls the mitochondrial transcription factor TFAM. TFAM is essential for mitochondrial DNA (mtDNA) maintenance and transcription of the 13 protein-coding genes of oxidative phosphorylation (OXPHOS). *Pdx1* deficiency in β-cells (as in MODY4 or conditional knockout mice) reduces TFAM expression and leads to mitochondrial DNA depletion, impaired respiratory chain function, and defective insulin secretion [57]. Notably, restoring TFAM expression in PDX1-deficient β-cells rescues mtDNA copy number, OXPHOS activity, ATP production and GSIS, highlighting how PDX1–TFAM signalling tightly couples mitochondrial bioenergetics to the nuclear β-cell identity program. A parallel mechanism of β-cell–mitochondrial coordination is exerted through TFB1M, a mitochondrial rRNA methyltransferase that stabilizes the 12S rRNA component of the mitoribosome [58]. Although originally described as a transcription factor, TFB1M is now known to be critical for post-transcriptional modification of mitochondrial ribosomes and the fidelity of mitochondrial protein synthesis. Mice with β-cell-specific *Tfb1m* deletion (*Tfb1m*^β−/−^) develop progressive insulin secretory failure and overt diabetes [59]. These islets exhibit markedly reduced levels of mitochondrial-encoded proteins, diminished ATP production, and impaired oxygen consumption [60,61]. Importantly, human carriers of a common *TFB1M* risk variant show reduced islet *TFB1M* expression and decreased insulin secretion [62], mirroring the mouse phenotype and providing direct evidence that even modest genetic variation in mitochondrial translation can predispose to β-cell failure and T2DM. Further, a study using ChIP-Seq profiling in human β-cell models (EndoC-βH1, primary islets, and hPSC-derived β-like cells) revealed that *HNF4α* target genes are enriched in pathways related to cytoskeletal remodelling and mitochondrial redox metabolism [63]. While actin dynamics and morphogenesis were common across cell types, insulin secretion and cAMP signalling were exclusively enriched in β-cells, suggesting *HNF4α*’s mitochondrial effects are embedded within β-cell specific transcriptional programs.

Beyond the direct transcriptional control of mitochondrial genes, emerging evidence indicates that mitochondrial metabolites themselves exert epigenetic regulation of β-cell identity genes through metabolic-epigenetic crosstalk. Acetyl-CoA, generated primarily through mitochondrial pyruvate oxidation and citrate export to the cytosol, serves as the obligate substrate for histone acetyltransferases (HATs). In β-cells, glucose stimulation increases mitochondrial acetyl-CoA production, which is then transported to the nucleus where it drives histone H3 acetylation at key β-cell gene loci, including *INS*, *PDX1*, and *MAFA* [64]. This acetylation opens chromatin structure and enhances transcription of genes essential for β-cell function and identity. Pharmacological or genetic disruption of ATP citrate lyase (ACLY), the enzyme that converts cytosolic citrate to acetyl-CoA, reduces histone acetylation and impairs insulin gene expression in β-cells [65]. Similarly, α-ketoglutarate (α-KG), a tricarboxylic acid (TCA) cycle intermediate, functions as an essential cofactor for the TET family of DNA demethylases and Jumonji-domain containing (JMJD) histone demethylases. α-KG availability directly influences the activity of these dioxygenases: under conditions of glucose stimulation, elevated mitochondrial α-KG export supports TET-mediated DNA demethylation at insulin enhancer regions and JMJD3-mediated removal of repressive H3K27me3 marks at β-cell maturity genes [66,67]. Conversely, when mitochondrial function is impaired, such as in models of *TFAM* deficiency or chronic glucolipotoxicity, α-KG levels decline, leading to hypermethylation of CpG islands and accumulation of repressive histone marks that silence β-cell identity programs [68]. This metabolic-epigenetic axis creates a direct link between mitochondrial bioenergetics and chromatin state, positioning mitochondrial dysfunction as not only a bioenergetic failure but also an epigenetic reprogramming event that drives β-cell dedifferentiation. Future studies employing stable isotope tracing combined with ChIP-seq and bisulfite sequencing in β-cells under metabolic stress will be essential to map the full scope of metabolite-driven epigenetic remodeling in diabetes pathogenesis.

Beyond transcriptional regulation, the translational capacity of β-cell mitochondria has emerged as a key regulator of β-cell performance. Recent evidence suggests that expression of mitochondrial ribosomal protein genes (MRPs) is downregulated in human T2DM islets [69], implicating impaired mitochondrial protein synthesis in disease pathogenesis. In particular, the mitoribosomal subunit *CRIF1* (also known as MRPL59) is partially reduced in diabetic islets. Mice with β-cell-specific haploinsufficiency of *Crif1* (*Crif1*^β+/−^) maintain normal glucose tolerance under basal conditions but exhibit selective loss of first-phase insulin secretion and accelerated islet failure when challenged by high-fat diet or aging [70]. Biochemically, *CRIF1* is essential for inserting nascent mitochondrial-encoded peptides into the inner mitochondrial membrane. Its reduction leads to decreased OXPHOS complex assembly and ATP production, consistent with the insulin secretion defect observed [71,72].

Other β-cell-expressed genes with mitochondrial roles have emerged from genetic studies. For instance, *CCDC66*, *LARS2*, *UCP2*, and *PPARGC1A* are implicated in mitochondrial dynamics, biogenesis, and coupling efficiency. *CCDC66*, though originally characterized for its role in ciliary function, has been found to regulate mitochondrial morphology in metabolically active tissues, including islets, where its loss leads to aberrant cristae structure and decreased respiratory capacity. *LARS2*, which encodes mitochondrial leucyl-tRNA synthetase, is required for accurate mitochondrial translation. Mutations in *LARS2* impair the incorporation of leucine into nascent mitochondrial proteins, thereby affecting the assembly and function of oxidative phosphorylation (OXPHOS) complexes. In β-cells, this can limit mitochondrial ATP production, leading to suboptimal insulin granule exocytosis [73,74]. UCP2 (uncoupling protein 2) modulates the proton gradient across the inner mitochondrial membrane, thus influencing the balance between ATP synthesis and heat generation. Although mild UCP2 expression can serve a protective antioxidant function, its overexpression has been shown to reduce ATP output and impair GSIS in rodent islets [75,76,77]. Notably, UCP2 levels are elevated in human T2DM islets, and polymorphisms in the UCP2 promoter region have been associated with altered T2DM risk and insulin secretion capacity in population studies [78,79,80]. Studies over the past decade indicate that UCP2 can function as a mitochondrial metabolite transporter, facilitating the export of C4 metabolites such as malate and aspartate. This activity influences redox balance rather than directly dissipating the proton gradient, highlighting a more nuanced role in fine-tuning substrate flow and redox coupling in β-cell metabolism [81].

**Figure 2:**
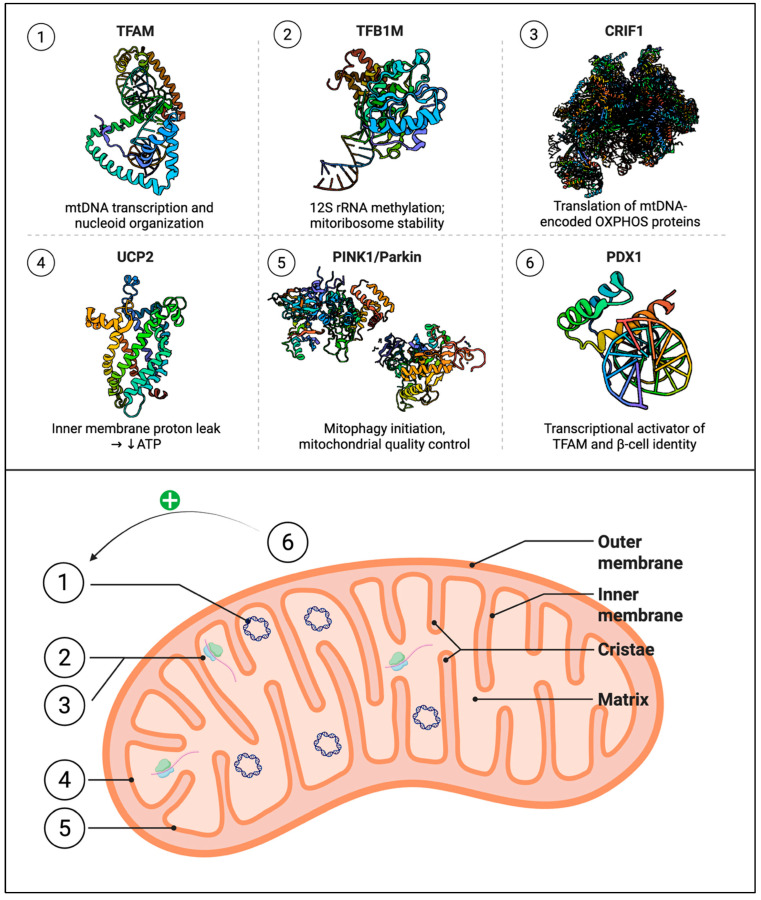
Structural features of key regulators of mitochondrial function in pancreatic β-cells. (1) *TFAM* (PDB: 3TMM) [106] bends mitochondrial DNA to promote transcription. (2) *TFB1M* (PDB: 6AJK) [58] methylates 12S rRNA; risk allele rs950994 may alter cofactor binding. (3) *CRIF1* (PDB: 3J7Y) [107] anchors mitoribosomes to the inner membrane to facilitate OXPHOS protein translation. (4) *UCP2* (PDB: 2LCK) [108] forms a proton leak channel that reduces ATP generation. (5) *PINK1* (PDB: 6EQI) [109] and *Parkin* (PDB: 6GLC) [110] initiate mitophagy by labeling depolarized mitochondria for degradation. (6) *PDX1* (PDB: 2H1K) [111] binds DNA to maintain β-cell identity and stimulate mitochondrial transcription factor *TFAM* (green plus sign ‘+’).

Despite these mechanistic insights, the physiological role of UCP2 in β-cells remains controversial, and several unresolved questions limit therapeutic translation. First, does UCP2 primarily function as a proton uncoupler, a metabolite transporter, or both, and does this depend on metabolic context? While biochemical studies demonstrate C4 metabolite transport activity [81], electrophysiological recordings in reconstituted systems also confirm proton leak conductance [82]. It remains unclear whether these activities occur simultaneously or are regulated by post-translational modifications such as glutathionylation, which has been shown to toggle UCP2 between transport and uncoupling modes [83]. Second, why do *Ucp2* knockout studies yield inconsistent results across different models? While *Ucp2*^−/−^ mice on an ob/ob background show improved insulin secretion and glycemic control [75], other studies report unchanged or even impaired β-cell function in *Ucp2* knockout mice on different genetic backgrounds or under different dietary conditions [84,85]. These discrepancies may reflect compensatory upregulation of other UCPs (UCP1, UCP3) or genetic background-specific differences in baseline mitochondrial coupling efficiency. Third, is the elevated UCP2 expression observed in human T2DM islets a cause or a consequence of metabolic dysfunction? Longitudinal studies tracking UCP2 levels during progression from prediabetes to overt diabetes are lacking, making it impossible to determine whether UCP2 upregulation is a primary driver of β-cell failure or a compensatory (albeit insufficient) antioxidant response to chronic oxidative stress [78,86]. Fourth, can UCP2 inhibition be safely targeted therapeutically without disrupting its cytoprotective antioxidant functions? Genipin, a natural UCP2 inhibitor, enhances insulin secretion in rodent models but has not been systematically evaluated in human islets or in vivo human studies, and its off-target effects on other mitochondrial proteins remain poorly characterized [87,88]. Resolving these controversies will require conditional, inducible β-cell-specific *Ucp2* knockout models combined with real-time imaging of both proton gradient dissipation and metabolite flux, as well as human islet studies correlating *UCP2* expression with functional outcomes across a range of metabolic states. Complementing these findings, transcriptional coactivator *PGC-1α* regulates both *NRF1* and *NRF2*, promoting transcription of nuclear-encoded genes required for ETC assembly [89]. In β-cells, *PGC-1α* must be tightly regulated. While it is crucial for adapting to increased energetic demands in other tissues, its overexpression in rodent islets suppresses insulin secretion and disturbs mitochondrial coupling efficiency. Genetic variants in *PPARGC1A*, such as the Gly482Ser polymorphism [90,91], have been linked to altered islet function, reduced insulin secretion, and increased T2DM risk in multiple populations [91,92,93,94,95,96,97,98]. While each factor contributes incrementally, they are best discussed together because their coordinated regulation is necessary for maintaining mitochondrial output during insulin secretion. Disruption at any point, whether through defective mitochondrial protein synthesis (LARS2), altered membrane coupling (UCP2), or dysregulated biogenesis (PPARGC1A), can weaken β-cell energetic capacity. Table 1 summarizes all known transcriptional regulators linking mitochondrial function, or lack thereof, to β-cell physiology, including but not limited to those discussed above. Meanwhile, Figure 2 summarizes the structural features of key transcriptional regulators.

## 4. Oxidative Stress, Nutrient Excess, and Consequences for β-Cell Failure

Chronic nutrient overload in diabetes drives β-cell mitochondrial stress. High glucose and free fatty acids increase substrate flux into mitochondria, hyperpolarizing the respiratory chain and generating excess superoxide [112,113]. Elevated ROS damages mitochondrial DNA, respiratory chain proteins, and lipid membranes [114]. In vitro, rodent β-cell lines (such as MIN6, INS-1E) and human islets exposed to glucolipotoxic conditions (glucose and palmitate) show elevated mitochondrial superoxide and H_2_O_2_ production [115,116,117], triggering apoptosis that is preventable by antioxidants [1]. In vivo, db/db and KK-Ay mice models show oxidative inactivation of key metabolic enzymes (e.g., aconitase, PDH) in β-cells, undermining ATP production [118,119].

Persistent oxidative stress also activates stress kinases (JNK, p38) that inhibit insulin gene expression and sensitize β-cells to apoptosis [120,121,122]. In a mouse model with β-cell-specific JNK activation (MKK7D), JNK impaired GSIS without affecting islet morphology or insulin content, indicating disrupted insulin signalling rather than cell loss [123]. However, this signalling defect included impaired AKT phosphorylation and PDX1 mislocalization, both essential for insulin gene expression, supporting JNK’s inhibitory role [124,125]. While JNK activation alone did not induce apoptosis, it sensitized β-cells to dysfunction, suggesting that in the presence of oxidative stress or inflammatory cues, JNK could contribute to β-cell apoptosis [123,126,127].

Under glucotoxic conditions, mitochondrial uncoupling often increases (via upregulation of UCP2 and related proteins), further reducing ATP yield and fuelling ROS as electrons leak [80]. This mechanism was confirmed in *Ucp2*-deficient mice, which exhibited higher islet ATP levels and potentiated insulin secretion in response to glucose. In another ob/ob mouse model, where UCP2 is upregulated, deletion of UCP2 restored first-phase insulin secretion and improved glycaemic control [75]. Reduced ATP generation means impaired closure of K_ATP_ channels and a weaker insulin response to glucose [128]. For example, in the β-cell *Tfam* KO model, glucose failed to depolarize mitochondria and raise ATP or Ca^2+^ levels [99] demonstrating how oxidative phosphorylation collapse translates to secretion failure [129]. Furthermore, oxidative stress can damage mtDNA, leading to mutations and deletions that further compromise mitochondrial function [130]. One study in diabetic human islets found evidence of mtDNA depletion and mutation accumulation with age, suggesting a feed-forward decline in mitochondrial capacity [131]. Though such mitochondrial DNA defects are not primary causes of common diabetes, they exacerbate β-cell dysfunction under chronic stress. Apart from nutrients, inflammatory cytokines (as in T1DM or late-stage T2DM) cause nitric oxide (NO) and ROS production in β-cells. NO reacts with superoxide to form peroxynitrite, which irreversibly damages mitochondrial electron carriers [132,133]. Indeed, proinflammatory cytokines induce β-cell mitochondrial fragmentation, loss of membrane potential, and mitophagy [33]. If the resulting damaged mitochondria are not removed, bioenergetic failure and cell death ensue.

New facets of the β-cell oxidative response should be noted. The NRF2 transcription factor is now recognized as a master antioxidant regulator in β-cells. Upon oxidative stress, NRF2 induces detoxifying enzymes to mitigate ROS damage [134]. Conversely, mitochondrial redox enzymes are themselves regulated by metabolic sensors. As noted above, SIRT3 activation increases MnSOD activity and lowers intracellular ROS. β-cells lacking SIRT3 have higher oxidative stress and poorer survival under glucolipotoxic conditions [135]. These cross-talk pathways suggest that antioxidant defenses are coupled to nutrient sensing. Emerging death pathways further expand this view. For instance, ferroptosis, an iron-dependent form of lipid peroxidation, can occur in β-cells when GPX4 is deficient, linking lipid oxidative stress to cell loss [136]. Incorporating NRF2 and sirtuin-mediated antioxidant control thus complements the classical view of ROS in β-cell failure.

The downstream effects of mitochondrial dysfunction on β-cell physiology are profound. The immediate impact is on GSIS [137]. Loss of mitochondrial ATP production precludes the closure of ATP-sensitive K^+^ channels, preventing membrane depolarization and Ca^2+^ influx that trigger insulin granule exocytosis [138]. This mechanism is well-illustrated by the *Tfam* knockout model: mutant β-cells showed reduced mitochondrial membrane hyperpolarization, blunted Ca^2+^ signalling and markedly lower insulin release to glucose [99]. Likewise, *Crif1*^β+/−^ mice lost the rapid first-phase insulin secretion that is normally triggered by the initial glucose rise [70]. In human T2DM islets, transcriptomic analyses reveal downregulation of virtually all mitochondrial gene sets (OXPHOS, translation, etc.) relative to non-diabetic islets, consistent with a global mitochondrial deficit that translates into secretion failure [139]. Beyond ATP, mitochondria generate coupling factors (e.g., NADPH, GTP, malonyl-CoA) that amplify GSIS through alternative signalling pathways [140]. For example, mitochondrial NADPH is necessary for the amplification pathway of insulin secretion and for sustaining ER redox balance [141]. Loss of these factors further impairs sustained insulin output during chronic glucose exposure. Even intermittent mitochondrial uncoupling (e.g., via UCP2) can lower ATP and dissipate metabolic signals, shifting β-cells towards a non-secretory state [142]. As mitochondrial impairment worsens, β-cells undergo apoptosis or necrosis.

**Figure 3:**
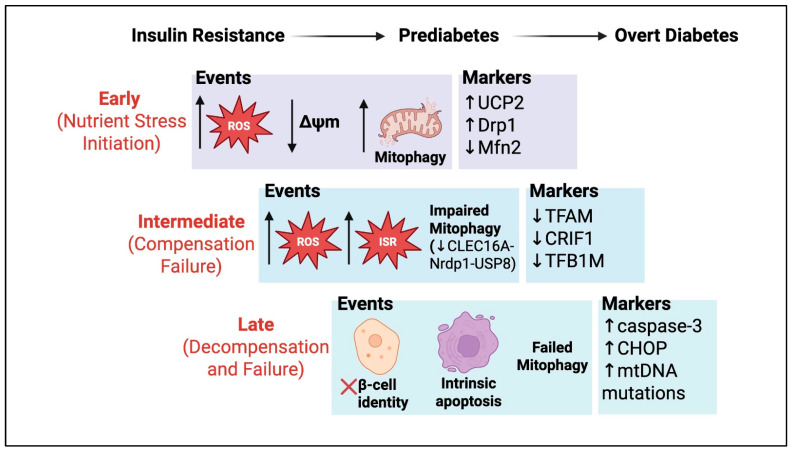
Progressive Stages of Beta-Cell Dysfunction in the Development of Type 2 Diabetes from Insulin Resistance Through Prediabetes to Overt Diabetes, Characterized by Early Nutrient Stress Initiation with Reactive Oxygen Species Generation, Mitochondrial Membrane Potential Changes, and Altered Mitophagy; Intermediate Compensation Failure with Persistent Reactive Oxygen Species, Integrated Stress Response Activation, and Impaired Mitophagy; and Late Decompensation and Failure with Loss of Beta-Cell Identity (red cross ‘X’), Intrinsic Apoptosis, and Failed Mitophagy. UCP2: uncoupling protein 2; DRP1: dynamin-related protein 1; MFN2: mitofusin 2; ROS: reactive oxygen species; Δψm: mitochondrial membrane potential; TFAM: transcription factor A mitochondrial; CRIF1: CREB regulated transcription coactivator 1; TFB1M: transcription factor B1 mitochondrial; ISR: integrated stress response; CLEC16A: C-type lectin domain family 16 member A; NRDP1: neuregulin receptor degradation protein 1; USP8: ubiquitin specific peptidase 8; CHOP: C/EBP homologous protein; mtDNA: mitochondrial DNA.

Chronic oxidative and nitrosative stress triggers mitochondrial permeability transition, characterized by cytochrome-c release and caspase activation [143]. Cytokine-treated β-cells demonstrate mitochondrial fragmentation and outer membrane permeabilization if mitophagy is blocked [33,144]. At the tissue level, this cell death reduces β-cell mass. Simultaneously, surviving β-cells often dedifferentiate or enter a senescent state, further reducing functional β-cell mass [145]. Notably, β-cells in long-standing diabetes show hallmarks of mitochondrial aging with fewer and damaged mitochondria, enlarged cristae and DNA deletions, analogous to aging neurons [146,147]. In non-human primate models, developmental exposure to maternal Western-style diet increased mitochondrial fragmentation in β-cells without altering maturity marker expression, implying that nutrient-driven morphological changes may occur independently of β-cell identity [148]. This aligns with observations in diabetes, where mitochondrial fragmentation and swelling often accompany β-cell failure. Inflammation and immune cell infiltration exacerbate this loss. Inflammatory mediators in both T1DM and T2DM impose additional stress on β-cell mitochondria (captured in Table 2 below). For example, sustained IL-1β exposure leads to iNOS induction and excess NO, which blocks complex IV and generates peroxynitrite in mitochondria [149]. This reinforces β-cell destruction in autoimmune diabetes. In T2DM, islet macrophage activation and NLRP3 inflammasome signals amplify mitochondrial stress [150,151]. Thus, mitochondrial dysfunction not only impairs insulin secretion but also sensitizes β-cells to immune and metabolic insults, creating a feed-forward loop of β-cell failure [24]. Table 2 summarizes examples of mitochondrial processes and proteins disrupted in diabetic β-cells, and their effects on insulin secretion, while Figure 3 shows mitochondrial events during insulin resistance, prediabetes, and overt diabetes. In general, impairments in mitochondrial dynamics or mitophagy lead to the accumulation of defective organelles, increased oxidative stress and cell death signals.

## 5. Therapeutics

Given the central role of mitochondria in β-cell health, strategies that protect or restore mitochondrial function hold therapeutic promise. Pharmacological and lifestyle interventions may target mitochondrial bioenergetics, redox balance and quality control in β-cells. Metformin is an established clinical therapy, considered by the majority as the first-line treatment for non-insulin dependent T2DM, with extensive evidence gathered from human studies across the years [154]. Further, in patients with new-onset T2DM, metformin has been shown to improve mitochondrial fitness in peripheral blood cells (PBCs), increase markers of mitophagy and reduce mitochondrial ROS and membrane polarization [155]. Although metformin’s primary site of action is the liver, these findings suggest that it may also exert beneficial effects on β-cell mitochondrial quality. Metformin activates AMP-activated kinase (AMPK) by inducing subtle changes in the ATP/ADP and ATP/AMP ratios, without significantly changing the total ATP levels [156]. Metformin acts primarily by activating AMPK in the liver at therapeutic doses (~40–80 μM in the portal vein, or ~50 mg/kg/day in high-fat diet (HFD)-fed mice). This activation suppresses gluconeogenesis, reduces hepatic glucose production and improves insulin sensitivity. In HFD-fed C57BL/6J mice, these doses of metformin enhance mitochondrial function by increasing complex I activity, ATP production, mitochondrial membrane potential, and by promoting mitochondrial fission. This supports mitophagy, maintains mitochondrial quality and increases nutrient oxidation. These effects are AMPK-dependent, in liver-specific AMPKα1/2 KO mice, metformin fails to improve glucose control or stimulate mitochondrial function. By contrast, suprapharmacological doses (≥500–1000 μM or 150–500 mg/kg/day), often used in in vitro studies or animal models, can suppress mitochondrial respiration by depleting cellular ADP levels, but this is not relevant to metformin’s clinical mechanism of action [1,157,158]. While hepatic AMPK activation is central to metformin’s glucose-lowering effects, AMPK also plays a critical role in pancreatic β-cells. To better understand this, researchers investigated the physiological consequences of completely ablating both catalytic isoforms of AMPK in β-cells [159]. Interestingly, in isolated islets from βAMPK double knockout (βAMPK.dKO) mice, GSIS was paradoxically enhanced. This was associated with increased K_ATP_ channel activity at low glucose concentrations, possibly due to altered subunit trafficking and more efficient granule translocation to the plasma membrane, likely resulting from the loss of AMPK-mediated inhibition via kinesin light chain phosphorylation [160,161,162]. However, in vivo studies revealed a different outcome: βAMPK.dKO mice exhibited markedly impaired insulin secretion following intraperitoneal glucose injection, leading to defective glucose homeostasis and hyperglycaemia [159]. This discrepancy highlights the context-dependent nature of AMPK signalling, suggesting that while β-cell-intrinsic AMPK constrains insulin secretion in vitro, its absence disrupts coordinated endocrine responses in vivo. Importantly, these defects were not due to reduced β-cell mass but may involve disrupted neuroendocrine regulation, as RIP.Cre-expressing neurons may influence β-cell function. Additionally, activation of AMPK specifically in β-cells (via βAMPK.CA transgenic mice) suppressed GSIS, consistent with pharmacological studies using metformin [1,157,163]. Beyond AMPK activation, other mitochondria-targeted interventions have emerged. Activating sirtuin–NAD^+^ axes is one promising strategy. Pharmacologic SIRT1/3 activators or NAD^+^ precursors (NR, NMN) have been shown to improve mitochondrial metabolism and β-cell function in preclinical diabetes models [164]. Some NAD^+^ boosters, such as NR and NMN, have entered early-phase clinical trials in humans for metabolic disorders, whereas specific SIRT1/3 activators remain at the preclinical stage [165,166,167,168,169]. Beyond that, antimiR therapy against miR-146a-5p rescued β-cell mitochondrial respiration and insulin secretion in vitro. These miRNA-based approaches have been investigated in non-human trials and have been showing positive results [170,171]. Enhancing mitophagy represents another approach [172]. In a β-cell *Clec16a*-knockout mouse with excessive Parkin activity and defective mitophagy, overexpressing the E3 ligase RNF41 restored Parkin balance and normalized mitochondrial morphology and insulin release [134]. Mitophagy modulation remains in its preclinical stages, with compounds such as urolithin A now advancing into early human studies for mitochondrial diseases [173,174]. Finally, novel cell-based therapies are on the horizon. Coculturing islets with mesenchymal stem cells markedly improved graft performance in vitro, partly because mesenchymal stem cells (MSCs) donated functional mitochondria to β-cells [175]. Indeed, co-culture of murine and human islets with adipose-derived MSCs improves β-cell survival, viability, and glucose-stimulated insulin secretion in vitro, and preconditioning via co-culture enhances engraftment and glycemic control in vivo after transplantation of marginal islet masses [176]. Mechanistically, these effects are attributed to mitochondrial transfer, paracrine secretion of trophic factors, gap-junctional contacts, and anti-inflammatory signaling, although direct visualization of mitochondrial donation in intact in vivo grafts remains limited [177,178,179]. Mitochondrial transfer and MSC-based approaches are currently in early preclinical stages, however MSC transplantation has been previously tested in clinical trials for diabetes [180,181,182,183]. Collectively, sirtuin/NAD^+^ boosters, miRNA inhibitors, mitophagy modulators, and mitochondrial transplantation illustrate the therapeutic breadth beyond classic antioxidants or UPR-targeting drugs.

Emerging evidence also implicates GLP-1 signalling in mitochondrial biogenesis and antioxidant defences in β-cells. GLP-1 increases mitochondrial calcium and ATP in MIN6 β-cells via cAMP-mediated activation of IP_3_ and ryanodine receptors. PKA mediates the IP_3_ pathway, while cAMP-GEFII regulates the ryanodine receptor pathway [184]. GLP-1 and exendin-4 upregulate *PGC-1α*, increasing mitochondrial mass, membrane potential and oxygen consumption [185]. Once upregulated, *PGC-1α* activates both *NRF1* and *NRF2*, promoting transcription of nuclear-encoded genes required for ETC assembly [89]. That said, impaired eIF2α phosphorylation disrupts ER proteostasis, leading to proinsulin accumulation and ER stress that contribute to β-cell dysfunction and death under metabolic stress. This ER stress exacerbates mitochondrial dysfunction by impairing mitochondrial biogenesis and ATP production, reducing the β-cell energy supply needed for insulin secretion. GLP-1R agonists mitigate ER stress through PKA-dependent modulation of the UPR, restoring eIF2α phosphorylation balance, which supports mitochondrial function by enhancing ATP synthesis and biogenesis [186,187]. Mitophagy activators and mild mitochondrial uncouplers both offer promising strategies to protect β-cells under diabetogenic stress [173,188,189,190,191]. Urolithin A, an activator, promotes Parkin-dependent mitophagy, facilitating the selective clearance of damaged mitochondria and maintaining mitochondrial integrity via CLEC16A-NRDP1-USP8-mediated signalling [173,190]. Mild uncouplers (e.g., niclosamide ethanolamine or BAM15) transiently depolarize mitochondrial membrane potential (Δψm), triggering mitophagy without causing bioenergetic collapse [192,193]. This controlled depolarization regulates mitochondrial turnover, reduces ROS and preserves β-cell function. Overall, therapeutic focus on β-cell mitochondria is promising but challenging. Table 3 summarizes examples of mitochondria-targeted interventions and their rationale.

## 6. Future Perspectives

Despite significant advances in our understanding of mitochondrial dysfunction in β-cells, several major gaps remain that offer compelling directions for future research and therapeutic development. Addressing these gaps will not only offer new mechanistic insight but may also redefine how we preserve β-cell health across the spectrum of diabetes.

### 6.1. Mitochondrial Heterogeneity and Spatial Organization

Current models often treat mitochondria in β-cells as a homogenous population, yet recent evidence from high-resolution imaging and single-organelle proteomics suggests substantial heterogeneity in mitochondrial structure, function and dynamics within individual cells [195]. Emerging tools such as high-sensitivity flow cytometry and spectral analyzers allow for the multiparametric profiling of individual mitochondria based on size, charge, membrane potential and redox state [196,197,198]. Evidence from other metabolically active cell types suggests that mitochondria demonstrate location-specific adaptations, supporting the possibility of functional subcompartmentalization in β-cells as well. This is particularly relevant in β-cells, where spatial and functional mitochondrial compartmentalization may directly impact insulin granule maturation, trafficking and secretion [199]. In parallel, organelle-targeted fluorescent reporters [200] and live-cell imaging systems [201,202] permit real-time tracking of mitochondrial dynamics in primary β-cells and stem-cell-derived islet models. For example, combining mito-targeted fluorescent proteins with cationic dyes like TMRE or Rhodamine 123 [203] can reveal mitochondrial subpopulations that exhibit differential membrane potential or calcium buffering, critical factors in GSIS. These tools can now be used to interrogate how mitochondria respond to oscillatory glucose patterns, identify mitochondrial subtypes associated with first-phase vs. second-phase insulin secretion, or even visualize the early signs of mitochondrial injury before overt β-cell failure. Recent single-organelle studies in other cell types have shown location-specific mitochondrial adaptations (e.g., perinuclear vs. cortical), a concept likely applicable to β-cells given their spatially constrained secretory architecture (reviewed in [195]). Notably, direct comparisons of isolated mitochondria with permeabilized-fiber and intact cell assays show that routine isolation alters mitochondrial morphology, increases ROS and mitochondrial permeability transition pore (mPTP) sensitivity, increases hydrogen peroxide production, and changes respiratory behavior and electron-transport stoichiometry [204]. In this context, mitochondria proximal to insulin granules may exhibit enhanced bioenergetic activity or calcium uptake, acting as localized ATP “hubs” for exocytosis, a hypothesis that could now be tested using modern spatial imaging and computational tools. It is conceivable that mitochondria proximal to insulin granules exhibit enhanced bioenergetic activity or calcium uptake, acting as localized ATP “hubs” for exocytosis. Future studies using mitochondrial pulse-chase labelling [205], mito-contact site imaging [206] and deep learning-assisted image analysis [207] in intact islets could uncover how these subdomains are established, maintained and lost during metabolic stress or in diabetes. Importantly, integrating such structural and functional datasets with single-cell transcriptomic and metabolomic profiles could enable the construction of a β-cell mitochondrial atlas that links mitochondrial diversity to functional resilience. Perhaps most importantly, mapping β-cell mitochondrial heterogeneity may lead to the discovery of functionally resilient subpopulations that resist glucolipotoxicity or cytokine insult. These could serve as biomarkers for β-cell robustness or as targets for sub-organelle-specific therapies. As these tools mature, a major goal will be to combine single-organelle analysis with single-cell transcriptomics and epigenomics to generate integrated β-cell mitochondrial atlases across diabetes progression. This approach promises to move beyond averages and reveal how mitochondrial individuality shapes β-cell fate and function.

### 6.2. Mitochondria-Derived Vesicles as Signaling Mediators

Mitochondria are not static entities but actively communicate with other organelles and the extracellular environment. One emerging vehicle for this communication is the mitochondria-derived vesicle (MDV), which can carry damaged proteins, lipids, or mtDNA fragments for degradation or immune signalling. MDV formation constitutes a rapid, selective mitochondrial response that can excise focal oxidative damage without committing the entire organelle to autophagic degradations [208]. When damage is extensive or disposal pathways are overwhelmed, canonical mitophagy is engaged and, if unresolved, mitochondrial outer membrane permeabilization and apoptosis follow [209]. These processes share common triggers and regulators (e.g., ROS, cardiolipin oxidation, PINK1/Parkin and mitochondrial-dynamics proteins), and failed MDV/mitophagy pathways can lead to extracellular release of mtDNA and oxidized lipids that activate innate immune sensors and amplify β-cell inflammation and death [210,211]. In β-cells, the role of MDVs is virtually unexplored, with only one study examining the transfer of inflammatory mitochondria via extracellular vesicles from M1 macrophages and subsequent induction of ferroptosis in β-cells in acute pancreatitis [212]. Building upon these initial observations, systematic characterization of MDV formation, and secretion dynamics in β-cells under metabolic stress could clarify whether MDVs represent an adaptive quality-control mechanism or a pathological signal of mitochondrial distress. Future research should assess whether MDV biogenesis is altered in metabolic disease, whether MDVs can serve as early biomarkers of mitochondrial stress, and whether modulation of MDV pathways can mitigate inflammatory responses or preserve mitochondrial integrity. Circulating MDVs could also serve as a non-invasive diagnostic tool to monitor β-cell mitochondrial stress in real time.

### 6.3. Rewiring the Integrated Stress Response

While the ISR is known to contribute to β-cell dedifferentiation [46], the possibility of selectively rewiring the ISR toward adaptive rather than maladaptive outcomes remains underexplored. Interventions that modulate ISR effectors, like selective ATF4 inducers or translational pause modulators [213,214], could allow β-cells to adapt to metabolic stress without losing identity. A future direction involves dissecting context-specific ISR outputs in β-cells using single-cell multiomics and determining whether certain ISR branches (e.g., ATF4-GADD34 vs. CHOP-driven apoptosis) can be decoupled therapeutically. Small molecules like ISRIB that enhance eIF2B activity may provide starting points for these investigations [215].

### 6.4. Precision Mitophagy Enhancement

Although mitophagy activators like urolithin A have shown promise, their broad action may not distinguish between healthy and dysfunctional mitochondria. Future efforts should focus on developing next-generation mitophagy enhancers that offer organelle-specific selectivity. One approach involves engineering synthetic Parkin recruiters [216] or PROTAC-like mitophagy tags [217] that are activated only under defined metabolic or redox conditions within β-cells. Alternatively, sensors of mitochondrial membrane potential that can be combined with drug-inducible degradation modules could permit real-time, spatially restricted mitophagy enhancement. The goal should shift from generalized mitophagy activation to precision mitochondrial pruning, maintaining a healthy mitochondrial network without compromising bioenergetic reserve.

### 6.5. Mitochondrial-Derived Peptides

Mitochondria encode not only canonical OXPHOS genes but also a growing family of mitochondria-derived peptides (MDPs) with signaling and metabolic functions. These small peptides, encoded by short open reading frames (sORFs) within the mitochondrial genome or bicistronic nuclear-mitochondrial transcripts, have emerged as novel regulators of cellular stress responses and metabolic homeostasis [218,219]. The best-characterized MDPs include humanin, MOTS-c (mitochondrial ORF of the 12S rRNA type-c), and the SHLP (small humanin-like peptide) family. While these peptides have demonstrated cytoprotective effects in neurons, cardiomyocytes, and skeletal muscle, their roles in β-cells remain poorly characterized despite compelling preliminary evidence. Humanin, a 24-amino acid peptide encoded within the 16S rRNA gene, has been shown to protect against ER stress-induced apoptosis by binding to the pro-apoptotic protein BAX and preventing its mitochondrial translocation [220,221]. In isolated islets, exogenous humanin administration reduces cytokine-induced β-cell death and preserves insulin secretion, suggesting it may function as an autocrine or paracrine survival factor under inflammatory stress [222]. MOTS-c, encoded in the mitochondrial 12S rRNA, acts as a metabolic regulator by enhancing AMPK activation and improving insulin sensitivity in peripheral tissues [223]. Intriguingly, MOTS-c can translocate to the nucleus under oxidative stress, where it interacts with antioxidant response elements to upregulate stress-defense genes [224]. Whether MOTS-c is produced in β-cells and whether it modulates GSIS or mitochondrial quality control pathways remains unknown. Mechanistically, MDPs may influence β-cell function through multiple routes: (1) direct modulation of mitochondrial respiration and ATP synthesis by stabilizing ETC complexes, as suggested by studies in cardiomyocytes [225]; (2) regulation of calcium handling by interacting with mitochondrial calcium uniporter (MCU) machinery, thereby fine-tuning the amplitude of calcium-driven insulin secretion [226]; and (3) activation of retrograde signaling pathways that enhance mitochondrial biogenesis through *PGC-1α* upregulation [227]. Importantly, circulating levels of humanin decline with age and are reduced in patients with T2DM, raising the possibility that MDP deficiency contributes to progressive β-cell failure [228]. Future research should employ ribosome profiling, mass spectrometry-based sORF discovery, and CRISPR screens to annotate the full β-cell MDP repertoire and functionally validate individual peptides in loss-of-function and gain-of-function models. Additionally, measuring MDP expression dynamics during the transition from compensated insulin resistance to overt diabetes may reveal whether MDPs serve as biomarkers of mitochondrial resilience or therapeutic targets to delay β-cell exhaustion.

### 6.6. Multi-Lineage Mitochondrial Dynamics in Intact Islets

Most mitochondrial studies in diabetes focus on β-cells in isolation, yet islets function as integrated micro-organs where β-cells constitute only 60–80% of the endocrine population, coexisting with α-cells, δ-cells, endothelial cells, pericytes and resident immune cells [229,230]. This architectural complexity suggests that mitochondrial resilience may vary dramatically across cell types, with the failure of one population potentially cascading into dysfunction of its neighbors through paracrine signaling or metabolic crosstalk [231,232]. α-cells face a unique metabolic challenge: secreting glucagon under low glucose conditions requires robust mitochondrial function, yet whether α-cell mitochondria fragment under chronic hyperglycemia (as β-cell mitochondria do) remains unexplored [233,234]. Similarly, δ-cells secrete somatostatin to regulate both insulin and glucagon release, and preliminary observations suggest their mitochondria operate at lower membrane potential with distinct cristae architecture compared to β-cells [235,236], though the functional implications for stress resistance are unknown. The possibility of mitochondrial communication between cell types adds another dimension to islet biology. Evidence from other tissues demonstrates that cells can transfer functional mitochondria via tunneling nanotubes or extracellular vesicles [237,238], raising the prospect that metabolically exhausted β-cells might receive organelle donations from neighboring cells. Conversely, damaged β-cell mitochondria may release danger signals, ROS, mtDNA fragments or inflammatory cytokines, that alter α-cell secretory behavior, perpetuating hormonal dysregulation [239]. Understanding how β-cell mitochondria adapt in concert with their neighbors will require spatially resolved approaches that preserve native islet architecture. Imaging mass cytometry, spatial transcriptomics and tissue clearing, combined with lightsheet microscopy, now permit visualization of mitochondrial networks across entire islets and multiple cell types simultaneously [240,241,242,243,244,245]. Such approaches may reveal paracrine resilience mechanisms or identify cell type-specific therapeutic targets that restore islet-wide metabolic coordination. The islet, viewed through this multi-lineage mitochondrial lens, emerges not as a collection of independent actors but as a metabolically coupled ensemble where mitochondrial health in one cell type directly shapes the fate of all others.

### 6.7. Circadian Regulation of Mitochondrial Function

Furthermore, the timing of metabolic cues is emerging as a novel modulator of mitochondrial function. The β-cell’s circadian clock influences fission–fusion balance and membrane potential. For instance, loss of the core regulator BMAL1 gene leads to excessive mitochondrial fission via upregulated FIS1, lowered membrane potential, and impaired insulin secretion [246]. Disruption of circadian rhythms by chronic hyperglycemia or poor sleep may therefore exacerbate mitochondrial fragmentation and dysfunction. New studies should examine how β-cell mitochondria behave across the circadian cycle, and whether time-restricted feeding or chronotherapy (timed drug delivery) can restore healthy mitochondrial dynamics. High-throughput “mitochondria-on-a-chip” assays could measure oscillations in OXPHOS activity or ROS production [247] over 24-h cycles in islets, illuminating chrono-metabolic vulnerabilities. Future perspectives on β-Cell mitochondrial research are summarized in Table 4 below.

Mitochondrial dysfunction remains a central driver of β-cell failure, but critical aspects, such as organelle heterogeneity, inter-organelle communication and adaptive stress responses, are still poorly understood. New tools now make it possible to study mitochondria at single-organelle resolution and in the spatial context of intact islets. The field should perhaps focus more on mechanistic and intervention-based work that tests how modulating mitochondrial quality, signalling and turnover can preserve β-cell function.

## Figures and Tables

**Table 1:** Transcriptional regulators linking mitochondrial function to β-cell physiology.

Gene Symbol	Function	Relevance to β-Cells/T2D	Reference
*TFAM*	Initiates and regulates mtDNA transcription	β-cell-specific knockout impairs insulin secretion and induces diabetes	[99]
*TFB1M*	Methylates mitochondrial 12S rRNA to stabilize ribosomes	Risk allele (rs950994) linked to reduced insulin secretion and mitochondrial dysfunction	[59]
*TFB2M*	Regulates transcription of mtDNA	Knockout causes reduced mtDNA, impaired ATP production, β-cell apoptosis	[100]
*PGC-1α*	Coactivates *NRF1*/2 for mitochondrial biogenesis	Overexpression suppresses insulin secretion; polymorphisms linked to β-cell dysfunction	[54,101]
*NRF1*	Regulates respiratory chain genes and *TFAM*	Polymorphisms associated with T2DM in Korean population	[102]
*PDX1*	Maintains β-cell identity and insulin expression	Controls mitophagy; reduced in *TFB2M*-deficient β-cells	[103]
*LARS2*	Catalyzes tRNA leucylation for mitochondrial translation	Genetic variation associated with T2DM susceptibility	[73]
*UCP2*	Modulates mitochondrial coupling and ATP production	Overexpression impairs insulin secretion; mildly elevated in T2DM islets	[80]
*FXN*	Facilitates iron-sulfur cluster assembly	Deficiency leads to β-cell apoptosis and mitochondrial ROS buildup	[104]
*PHB2*	Maintains mitochondrial cristae integrity	Knockdown impairs GSIS and causes β-cell loss	[105]

**Table 2:** Key mitochondrial processes and quality control pathways disrupted in diabetes with mechanistic insights into β-cell dysfunction metabolic consequences and supporting experimental evidence.

Process/Factor	Dysfunction in Diabetes	Consequences for β-Cell	Example/Ref.
mtDNA maintenance (*TFAM*)	Downregulation or knockout of *TFAM*, mtDNA depletion	Collapse of OXPHOS, failed ATP production, impaired Ca^2+^ signalling, reduced GSIS; progressive β-cell loss	*Tfam* β-cell KO mice [57,99]
Mitoribosomes (*CRIF1*, *TFB1M*)	Reduced expression or mutation of *CRIF1* (MRPL59) or *TFB1M* in β-cells	Impaired mitochondrial translation, lower ATP/O_2_ consumption, reduced first-phase insulin release; β-cell failure under stress	*Crif1^β^*^+/−^ mice [70]; *Tfb1m*^β−/−^ mice [59]
Mitophagy (PINK1/Parkin, *CLEC16A*)	Impaired ubiquitin signalling (e.g., *CLEC16A* deficiency) or mitophagy blockade	Accumulation of damaged mitochondria, decreased respiration, increased apoptosis [33]	CLEC16A-NRDP1-USP8 complex inactivation [40]
Oxidative phosphorylation (respiratory chain)	Inhibition by ROS or nutrient stress (e.g., UCP2 upregulation, calcium overload)	Reduced ATP synthesis, depolarized mitochondria, blunted insulin secretion; eventual β-cell apoptosis [99]	UCP2 upregulation, ROS damage [75,83,152]
ER–mitochondria coupling	Chronic ER stress, disrupted ER Ca^2+^ handling	NADPH imbalance, disrupted protein folding, feed-forward mitochondrial damage; impaired insulin biosynthesis	ER stress in T2DM β-cells [8,153]

**Table 3:** Current therapeutic approaches and experimental strategies to restore beta-cell mitochondrial function and survival.

Strategy/Agent	Mechanism	Evidence/Effect	Stage of Therapeutic Approach	References
Metformin	Activates AMPK, enhances mitophagy, reduces mitochondrial ROS	Improves mitochondrial fitness and insulin secretion; increased mitophagy markers	In vitro	[160,161,162]
GLP-1 receptor agonists	Enhance cAMP/PKA signalling; upregulate biogenesis and survival	Promote β-cell proliferation/survival and possibly mitochondrial biogenesis	In vitro	[184,185]
Gene therapy (*TFAM*, *TFB1M*)	Restore expression of mitochondrial transcription factors	*TFAM* re-expression rescues insulin secretion in PDX1-deficient islets	In vitro and in vivo	[57]
Mitophagy activators	Stimulate PINK1-Parkin pathway or CLEC16A complex	Increased clearance of damaged mitochondria; improves β-cell survival (preclinical)	In vitro and in vivo	[33]
Lifestyle (diet, exercise)	Reduce metabolic stress; induce biogenesis	Improves whole-body insulin sensitivity; may enhance β-cell mitochondrial function indirectly (via lower glucose/weight)	Clinical guideline	[194]

**Table 4:** Future Directions in β-Cell Mitochondrial Research Highlighting Key Knowledge Gaps, Innovative Tools, and Therapeutic Opportunities to Preserve Cellular Function in Diabetes.

Focus Area	Key Knowledge Gap/Question	Emerging Tools & Approaches	Proposed Future Directions/Therapeutic Potential
Mitochondrial Heterogeneity	Current models treat β-cell mitochondria as uniform, despite evidence of structural and functional diversity.	High-sensitivity flow cytometry, spectral analyzers, organelle-targeted fluorescent reporters, live-cell imaging, mito-targeted dyes (TMRE, Rhodamine 123), deep learning image analysis.	Characterize mitochondrial subpopulations linked to insulin secretion phases. Build β-cell mitochondrial atlases integrating imaging, transcriptomics, and metabolomics. Identify functionally resilient mitochondrial subtypes resistant to metabolic stress.
Organelle Communication via Mitochondria-Derived Vesicles (MDVs)	Role of MDVs in β-cells is virtually unexplored.	MDV formation and secretion assays, extracellular vesicle profiling.	Define whether MDVs act as adaptive or pathological signals. Explore MDVs as biomarkers of mitochondrial stress or therapeutic targets. Assess MDVs as non-invasive indicators of β-cell mitochondrial health.
Integrated Stress Response (ISR) Modulation	Unclear how to selectively shift ISR from maladaptive to adaptive signaling in β-cells.	Single-cell multiomics, ISR modulators (e.g., ATF4 inducers, ISRIB).	Dissect ISR branch-specific effects (adaptive vs. apoptotic).—Develop ISR-targeted therapies preserving β-cell identity and stress tolerance.
Precision Mitophagy Enhancement	Current mitophagy activators lack selectivity between healthy and damaged mitochondria.	Synthetic Parkin recruiters, PROTAC-like mitophagy tags, voltage-sensitive degradation sensors.	Design mitochondria-specific mitophagy enhancers that act only under defined redox/metabolic states. Aim for precision mitochondrial pruning to maintain functional networks.
Mitochondria-Derived Peptides (MDPs)	Poorly characterized in β-cells despite known roles in other tissues.	Ribosome profiling, mass spectrometry-based sORF discovery, CRISPR functional screens.	Annotate β-cell mitoproteome to identify novel MDPs. Explore MDPs as regulators of insulin secretion, oxidative stress, and UPR. Develop MDP-based therapeutic peptides.
Multi-Lineage Mitochondrial Interactions in Islets	Focus has been primarily on β-cells, neglecting α- and δ-cell mitochondrial adaptations.	Spatial transcriptomics, imaging mass cytometry, tissue clearing with mitochondrial dyes.	Map mitochondrial dynamics across all islet cell types. Investigate paracrine mitochondrial resilience mechanisms.
Circadian Regulation of Mitochondrial Function	Temporal control of mitochondrial dynamics in β-cells is understudied.	Mitochondria-on-a-chip systems, circadian models, chrono-metabolic assays.	Examine mitochondrial behavior across circadian cycles. Assess impact of circadian disruption on β-cell mitochondrial health. Explore chronotherapy and time-restricted feeding to restore function.
Integrative Framework for β-Cell Mitochondrial Research	Lack of unified view connecting mitochondrial quality, signaling, and turnover to β-cell health.	Single-organelle and spatial imaging integrated with omics data.	Combine mechanistic and therapeutic studies. Test interventions targeting mitochondrial quality control and signaling to preserve β-cell function in diabetes.

## Data Availability

No new data were created or analyzed in this study.
